# Supplementary material for: Viral fusion proteins of classes II and III recognize and reorganize complex biological membranes
Source: Commun Biol. 2025 May 9;8:717. doi: 10.1038/s42003-025-08040-9 (PMC12062360; doi:10.1038/s42003-025-08040-9)
Supplement: Supplementary file 5 — Reporting Summary [file 42003_2025_8040_MOESM5_ESM.pdf]

nature portfolio

Corresponding author(s): Jochen S Hub

Last updated by author(s): Mar 25, 2025

Reporting Summary

Nature Portfolio wishes to improve the reproducibility of the work that we publish. This form provides structure for consistency and transparency in reporting. For further information on Nature Portfolio policies, see our Editorial Policies and the Editorial Policy Checklist.

Statistics

For all statistical analyses, confirm that the following items are present in the figure legend, table legend, main text, or Methods section.

n/a	Confirmed	
☐	☒	The exact sample size (n) for each experimental group/condition, given as a discrete number and unit of measurement
☒	☐	A statement on whether measurements were taken from distinct samples or whether the same sample was measured repeatedly
☒	☐	The statistical test(s) used AND whether they are one- or two-sided<br>*Only common tests should be described solely by name; describe more complex techniques in the Methods section.*
☒	☐	A description of all covariates tested
☒	☐	A description of any assumptions or corrections, such as tests of normality and adjustment for multiple comparisons
☐	☒	A full description of the statistical parameters including central tendency (e.g. means) or other basic estimates (e.g. regression coefficient) AND variation (e.g. standard deviation) or associated estimates of uncertainty (e.g. confidence intervals)
☒	☐	For null hypothesis testing, the test statistic (e.g. F, t, r) with confidence intervals, effect sizes, degrees of freedom and P value noted<br>*Give P values as exact values whenever suitable.*
☐	☒	For Bayesian analysis, information on the choice of priors and Markov chain Monte Carlo settings
☒	☐	For hierarchical and complex designs, identification of the appropriate level for tests and full reporting of outcomes
☒	☐	Estimates of effect sizes (e.g. Cohen's d, Pearson's r), indicating how they were calculated

Our web collection on statistics for biologists contains articles on many of the points above.

Software and code

Policy information about availability of computer code

Data collection	All simulations were carried out using publicly available open source code GROMACS, versions 2020.2
Data analysis	Analysis were performed using inbuilt GROMACS 2020.2 tools, VMD 1.9.4 and Clustal Omega. Data plotted using matplotlib

For manuscripts utilizing custom algorithms or software that are central to the research but not yet described in published literature, software must be made available to editors and reviewers. We strongly encourage code deposition in a community repository (e.g. GitHub). See the Nature Portfolio guidelines for submitting code & software for further information.

Data

Policy information about availability of data

All manuscripts must include a data availability statement. This statement should provide the following information, where applicable:

- Accession codes, unique identifiers, or web links for publicly available datasets
- A description of any restrictions on data availability
- For clinical datasets or third party data, please ensure that the statement adheres to our policy

All data are available in the main text and/or the sup- plementary materials. Coarse-grained and all-atom structure files, topologies, parameter files, backmapping files, and other related files required to reproduce the simulation data are available at Zenodo: https://doi.org/10.5281/zenodo.7945458. Source data for the graphs are provided as a Source data file. Additional data that support the findings of this study are available from the corresponding author upon request.

Research involving human participants, their data, or biological material

Policy information about studies with human participants or human data. See also policy information about sex, gender (identity/presentation), and sexual orientation and race, ethnicity and racism.

Reporting on sex and gender	No human participants were involved in this study.
Reporting on race, ethnicity, or other socially relevant groupings	No human participants were involved in this study.
Population characteristics	No human participants were involved in this study.
Recruitment	No human participants were involved in this study.
Ethics oversight	No human participants were involved in this study.

Note that full information on the approval of the study protocol must also be provided in the manuscript.

Field-specific reporting

Please select the one below that is the best fit for your research. If you are not sure, read the appropriate sections before making your selection.

☒ Life sciences ☐ Behavioural & social sciences ☐ Ecological, evolutionary & environmental sciences

For a reference copy of the document with all sections, see nature.com/documents/nr-reporting-summary-flat.pdf

Life sciences study design

All studies must disclose on these points even when the disclosure is negative.

Sample size	The membrane patches were large enough to exclude periodic boundary artifacts. The simulation time (coarse-grained conventional simulations: 45 µs per membrane composition, All-atom simulations: total simulation time of 40 µs and coarse-grained umbrella sampling simulations: 500ns per umbrella window) and the number of independent simulations was chosen large enough to exclude any sampling issues or reproducibility issues. In umbrella sampling simulations the error bars were on the order of only few kilojoule per mole.
Data exclusions	No data were excluded from the analyses.
Replication	All data in this manuscript have been successfully reproduced as outlined in the respective figure legends and/or the methods section. All attempts of replication simulations results were successful.
Randomization	Randomization do not change the outcome of our simulations.
Blinding	Blinding was not relevant to this study and would not have changed the results.

Reporting for specific materials, systems and methods

We require information from authors about some types of materials, experimental systems and methods used in many studies. Here, indicate whether each material, system or method listed is relevant to your study. If you are not sure if a list item applies to your research, read the appropriate section before selecting a response.

Materials & experimental systems

n/a	Involved in the study	
☒	☐	Antibodies
☒	☐	Eukaryotic cell lines
☒	☐	Palaeontology and archaeology
☒	☐	Animals and other organisms
☒	☐	Clinical data
☒	☐	Dual use research of concern
☒	☐	Plants

Methods

n/a	Involved in the study	
☒	☐	ChIP-seq
☒	☐	Flow cytometry
☒	☐	MRI-based neuroimaging

Plants

Seed stocks	Not relevant for this study
Novel plant genotypes	Not relevant for this study
Authentication	Not relevant for this study
